# Regulation of innate immunity by liquid–liquid phase separation: a focus on veterinary viruses

**DOI:** 10.3389/fmicb.2025.1667745

**Published:** 2025-09-10

**Authors:** Zibo Zhou, Qingyang Yuan, Kewei Fan, Teng Huang

**Affiliations:** ^1^College of Animal Science and Technology, Guangxi University, Nanning, China; ^2^Fujian Provincial Key Laboratory for Prevention and Control of Animal Infectious Diseases and Biotechnology, Longyan University, Longyan, China; ^3^Guangxi Key Laboratory of Animal Breeding, Disease Control and Prevention, Guangxi University, Nanning, China; ^4^Guangxi Zhuang Autonomous Region Engineering Research Center of Veterinary Biologics, Guangxi University, Nanning, China

**Keywords:** innate immunity, liquid–liquid phase separation, biomolecular condensates, viral infections, domestic animals

## Abstract

For most pathogenic viruses, maintenance of their active life cycles requires a diverse array of strategies that efficiently mobilize the limited resource from host cells. Intriguingly, it remains elusive on how the essential building blocks are recruited and assembled to produce a large number of infectious virions within a crowded intracellular compartment, while the host innate immune constituents are deliberately excluded from this viral niche. Recently, emerging evidence has suggested that the intricate interplay between host and virus can invariably be modulated by a general physicochemical basis, known as liquid–liquid phase separation (LLPS). This mini-review outlines the mechanisms underlying LLPS that regulate the animal virus replication and finetune the innate immune signaling network, with a particular focus on manipulation of LLPS by veterinary viruses to antagonize the host innate immunity. With increased understanding of how viruses hijack LLPS for their persistence and immune evasion, more effective and targeted antivirals or therapeutics will be developed to prevent the enormous losses of domestic animals caused by viral infections.

## 1 Introduction

Despite the success of vaccines in containing animal viral diseases, emerging and re-emerging viruses remain a significant threat to livestock production and animal welfare worldwide (Yadav et al., 2020). To develop more effective antivirals and therapeutics, continuous research efforts are needed to exploit the mechanisms that activate robust immune responses, while inhibit the critical steps of virus life cycle (Strumillo et al., 2021). Numerous theories have been proposed to define and interpret the specific molecular interactions between viral components and host factors (Mukherjee and Bagchi, 2024). However, these assumptions fail to fully explain the precise spatiotemporal regulation observed in both viral assembly factories and compartmentalized antiviral responses by the host (De Castro et al., 2021; Dugied et al., 2025).

Liquid–liquid phase separation (LLPS) is a basic physicochemical process found in nearly all cell types. LLPS empowers the formation of membraneless organelles that aggregate different functional biomolecules to promote the amplification of intracellular signaling. Phase transition of biomolecules is tightly regulated by LLPS in a concentration-dependent manner (Shin and Brangwynne, 2017). It has been increasingly appreciated that virus and host are competing for LLPS to gain their own benefits, and LLPS indeed has become a major coordinator that maintains the subtle homeostasis between immune activation and tolerance. As an important pathogen for poultry, Newcastle disease virus (NDV) has been shown to employ LLPS for establishing viral replication compartments through condensation of its NP and P proteins (Wang et al., 2023). In contrast, stress granules (SGs) mediated by LLPS in host cells prevent virus from accessing essential building components (Sun et al., 2017).

Compared to humans and model animals, less is known about the roles of LLPS in regulating veterinary species and viruses, but there is sufficient evidence to support more investigations in this area. The higher expression of heat shock protein HSPA8 is correlated with elevated temperature in animals. Interestingly, HSPA8 enhances the stability of inflammation-related proteins and autophagic process by LLPS (Miao et al., 2023), which might be beneficial for clearance of invading bacteria and viruses. Moreover, poultry cells have relatively higher content of proteins (Karaffová et al., 2025). Some protein families, such as Dact1 that serves an important modulator of myogenesis via the Wnt signaling, are expressed more abundantly than mammalian cells and likely undergo LLPS as predicted by its long intrinsically disordered regions (Contriciani et al., 2021). Thus, in theory poultry cells might contain more scaffold targets for LLPS to occur. From the translational perspective, development of targeted antivirals exclusive for food-producing animals will be feasible, if the dynamic nature of phase-separated biomolecules involved in virus-specific cellular processes can be harnessed and druggable, which is helpful to mitigate the burden of broad-spectrum antivirals to the public health.

This mini-review begins with an introduction of cellular compartments powered by LLPS to support replication and proliferation of veterinary viruses, and describes the conserved mechanisms of LLPS that control innate immune signaling. By summarizing the strategies that viruses disrupt immune biomolecular condensates, we posit the approaches on how the LLPS-mediated interactions between host and virus can be leveraged to develop more effective antivirals and therapeutics against viral infections.

## 2 LLPS enhances replication and proliferation of animal viruses

As obligatory intracellular parasites, viruses have adapted to the harsh cellular environment by efficiently recruiting essential building blocks (e.g., nucleic acids and proteins) to generate an exponential number of progenies. The active sites of virus replication can be microscopically observed in the nucleus or in the cytoplasm depending on the characteristic properties of virus genome. Various types of virus-containing subcellular compartments have been identified, including nuclear viral replication compartments (VRCs), cytoplasmic viral factories (VFs) and inclusion bodies (IBs). Surprisingly, their formation has consistently been associated with the induction of LLPS (Table 1).

**Table 1:** LLPS-mediated virus-host interactions.

Mode of interaction	Virus	Viral protein or host pathway	Mechanism
Hijacking LLPS for viral replication	Herpesviruses (HSV-1, PRV, EHV-1)	ICP4, IE	Formation of nuclear VRCs to enhance viral genome replication
	SARS-CoV-2	N/M	Assembly of VF-like condensates with RNA and M protein for virion production
	Influenza A viruses (IAV, AIV)	vRNP	LLPS with Rab11 to form cytoplasmic vRNP condensates supporting assembly
	PEDV	NSP3, NSP4, NSP6	Recruitment of host YIPF5 to facilitate DMV formation via LLPS
	NDV	NP/P	Core drivers of cytoplasmic inclusion body (IB) formation for replication
	Rotavirus	vNSP2	LLPS-driven targeting to lipid droplets for optimized genome replication
	RABV	P	Formation of Negri bodies as replication hubs by thermo-responsive LLPS
	MCMV	pE1/pM25/pM48.2/pM57	Participation in the formation of viral replication compartments
Induction of antiviral innate responses by LLPS	HSV-1	cGAS-STING pathway	Potentiation of IFN signaling through enhanced cGAMP synthesis and DNA protection
	Influenza A viruses	dsRNA-PKR-eIF2α-SGs axis	Prevention of excessive immune activation and promotion of interferon production via SG assembly
	PRV	PML nuclear bodies (PML-NBs)	Suppression of viral gene transcription through recruitment of antiviral proteins
	Rotavirus	Inflammasomes	Induction of pyroptosis and cytokine release against viral infections via caspase-1 activation
Disruption of LLPS machinery by viruses to evade innate immunity	ASFV	DP71L/QP383R	DP71L disrupts STING-TBK1 interactions; QP383R inhibits cGAS DNA binding and LLPS
	MDV	Meq	Steric hindrance of TBK1/IRF7 recruitment to STING complexes
	FMDV	L^Pro^	Cleavage of G3BP1/2 to disrupt stress granules
	PRV	IE180	Nuclear relocation of G3BPs to inhibit SG formation
	PRRSV	nsp1	Suppression of TRIM19-dependent PML-NB formation
	CIAV	VP3 (apoptin)	SUMOylation-dependent targeting of PML-NBs to disrupt antiviral function
	JEV	Not determined	Selective destabilization of PML isoforms

### 2.1 Nuclear viral replication compartments

An intrinsically disordered protein (IDP) is defined as a folded protein that is completely or partially devoid of a unique fold in isolation in solution, and exhibits limited secondary structure (Trivedi and Nagarajaram, 2022). Viral IDPs (vIDPs) induce protein condensation via LLPS. Examples include rabies virus (RABV) or vesicular stomatitis virus (VSV) phosphoprotein P (Nikolic et al., 2017; Heinrich et al., 2018) and herpesviral proteins such as ICP4 (Seyffert et al., 2021) that are linked to animal infectious diseases.

DNA viruses create nuclear viral replication compartments (VRCs), which are the phase-separated products and function as the platform for generating progeny virions. Through the properties attributed to them, these compartments promote the spatial organization of viral processes and control virus-host interactions (Charman and Weitzman, 2020). ICP4, an immediate-early protein (IEP) family member and viral intrinsically disordered protein (IDP), plays a crucial role in viral replication and has been implicated in driving LLPS within herpes simplex virus type 1 (HSV-1) VRCs (Seyffert et al., 2021). ICP4 and its homologs are broadly conserved in alphaherpesviruses with the ICP4 [the IE180 of pseudorabies virus (PRV), IE of equine herpesvirus type 1 (EHV-1) and homologs in infectious laryngotracheitis virus (ILTV) exhibiting extensive sequence homology (Wu and Wilcox, 1991; Anderson et al., 1992; Johnson et al., 1995)]. It is likely that these proteins have similar functions, as PRV mutants deficient in the *IE180* gene failed to generate new viral genomes, synthesize immediate early, early or late viral proteins and assemble infectious virions (Wu et al., 2014), and EHV mutants deficient in their IE protein only replicate in IE13.1 cells (Garko-Buczynski et al., 1998). In HSV-1, the nuclear form of VP22 specifically co-localizes with ICP4-enriched viral replication compartments (Pomeranz and Blaho, 1999), whereas EHV VP22 increases ICP4 synthesis after transcriptional activation (Okada et al., 2018). Antisense oligodeoxynucleotides against the putative start codon region of MDV ICP4 mRNA inhibit MSB-1 cell proliferation (Xie et al., 1996). Collectively, these findings indicate that the mechanism of IE protein-driven LLPS in NFAT-activated nuclear VRC formation is widely conserved among animal alphaherpesviruses. The proteins pE1, pM25, pM48.2, and pM57 of murine cytomegalovirus (MCMV) are simultaneously involved in VRCs. This process occurs in both the cytoplasm and the nucleus, functioning as a compartmentalization mechanism during the nuclear phase of replication in the viral replication cycle (Mahmutefendić Lučin et al., 2023).

### 2.2 Cytoplasmic viral factories

The N protein of SARS-CoV-2 can undergo phase separation with viral RNA and the membrane-associated protein M, forming structures resembling viral factories (VFs). In the virion, the N protein forms vRNPs with RNA; these vRNPs are arranged in a specific shell-like configuration within the virus membrane. The interaction between the N protein and the M protein may facilitate the recruitment of vRNPs into developing virus particles, thereby promoting viral assembly (Lu et al., 2021). It is noteworthy that the vRNP of influenza A virus (IAV) and the host protein Rab11 form membraneless liquid organelles in the cytoplasm. These structures can assemble without requiring RNA interactions between different vRNP segments. While not involved in immune evasion, they support viral assembly (Alenquer et al., 2019), exhibiting features of VFs. Although IAV is not typically classified as a veterinary virus, its vRNP core proteins containing NP and RNA are evolutionarily conserved (Hu et al., 2017; Li et al., 2021). This suggests that the core vRNP or similar structures in species-specific IAVs, including avian influenza virus (AIV), swine influenza virus (SIV), and bat influenza A virus (BAT IAV), may utilize a consensus LLPS mechanism to form VFs or assembly centers. These dynamic and efficient viral replication sites not only concentrate the components required for viral genome replication and transcription, but may also spatially isolate or shield viral nucleic acids. NP inhibitors (such as F66, naproxen, RK424, etc.) may exhibit good inhibitory effects against these viruses, although challenges related to the poor bioavailability of some drugs need to be addressed (Hu et al., 2017). Additionally, porcine epidemic diarrhea virus (PEDV) uses the host factor YIPF5, whose knockdown impairs viral replication and inhibits PEDV infection. YIPF5 interacts with viral nonstructural proteins (NSP) 3, 4, and 6 to facilitate double-membrane vesicle (DMV) formation, similar to VFs. YIPF5 knockdown disrupts the interaction between NSP3 and NSP4, impairing DMV formation (Guo et al., 2025). These findings suggest YIPF5 as a key host factor in PEDV infection and highlight its potential as an antiviral target.

### 2.3 Inclusion bodies

Inclusion bodies (IBs) are membraneless organelles that arise via viral LLPS and localize to a number of cellular compartments to support viral replication. The viral IBs also reorganize cellular architecture and cytoplasmic contents, including the cytoskeleton, a process that may disrupt immune response pathways, then facilitating viral assembly and budding (Wu et al., 2022).

The capsid of the zoonotic arthropod-borne Getah virus (GETV) undergoes LLPS droplets with the viral genomic RNA (gRNA) *in vitro* and forms cytoplasmic puncta to promote viral assembly. Two regions of the GETV gRNA (1–4,000 and 5,000–8,000 nucleotides) have been identified to enhance the *in vitro* formation of capsid protein (CP) droplets. The lysine-rich linker region within CP is crucial for phase separation. The ability of CP to undergo LLPS is nearly abrogated, when the lysine residues are substituted with the glutamic acids in the (K/E) mutant (Sun et al., 2024).

NDV can also form cytoplasmic IBs by LLPS, with the NP and P proteins serving as core drivers of condensate formation. In this process, the N terminal domain, N core region of NP and the C terminus of P play important roles (Sun et al., 2017). Considering NDV has oncolytic potential that specifically kills malignant cells (Liao et al., 2024), we speculate that their oncolytic specificity may correlate with strict LLPS regulation. In this regard, tumor cells are frequently characterized by the overexpression of IDR-containing proteins (e.g., oncogenic fusion proteins) that could act as scaffolds for NDV IBs and would therefore stabilize NP-P condensates and promote viral replication exclusively in malignant cells.

Rotavirus is a double-stranded RNA virus, and during replication forms cytoplasmic electron-dense inclusions that are sites for dsRNA synthesis and virion assembly (Geiger et al., 2021; Papa et al., 2021). Phosphorylation of NSP2 is essential for triggering LLPS-driven viral factory assembly. CK1α kinase phosphorylates NSP2 at serine 313 (S313), causing its transition from the dispersed cytoplasmic state (dNSP2) to the virus factory-localized state (vNSP2). Phosphorylated vNSP2 co-localizes with lipid droplet marker P-PLIN1 as early as 4 hpi, indicating that it targets the lipid droplet interface and may recruit host lipid metabolism components through phase separation. The hydrophobic lipid droplet surface promotes vNSP2 polymerization and phase separation. Virus-induced lipid droplet biogenesis (e.g., PLIN1 phosphorylation) optimizes the LLPS microenvironment, forming condensates favorable for viral genome replication. NSP2 phosphorylation acts as a “molecular switch” that coordinates phase separation of rotavirus proteins and host factor recruitment to optimize viral replication (Criglar et al., 2020).

RABV induces membraneless liquid organelles (Negri bodies) in the cytoplasm of the host for replication (Zhang et al., 2014), and its phosphoprotein (P) acts as a scaffold driven by LLPS to promote the assembly of Negri bodies (Nevers et al., 2022). Negri bodies contain the viral genome, antisense genome, and mRNA. Detection with BrUTP assay demonstrates the occurrence of viral transcription and replication in this compartment (Lahaye et al., 2009). This LLPS is a thermo-responsive process. In aqueous solutions at pH 7.5, RABV P undergoes autonomous phase separation above the lower critical solution temperature (LCST), modulated by salt concentration (Bouchama et al., 2025). Since RABV spreads through Negri bodies, understanding the dynamics of LLPS under specific temperature and salt conditions might help develop novel treatments for this still incurable disease.

Taken together, as viruses hijack host immune-driven LLPS to promote their replication, precisely targeted therapeutic strategies, such as disrupting virus-specific LLPS-driving domains or employing proteolytic degradation systems could be developed; meanwhile, combinatorial approaches that integrate LLPS modulators with immune agonists, such as STING activators VAX014 (Nelson et al., 2025), may synergistically enhance antiviral efficacy through multi-mechanistic interventions.

## 3 The conserved LLPS machinery responsible for antiviral innate immunity

LLPS has an important role in activation of innate immune signaling. It is marked by the selective enrichment and exclusion of certain proteins. At present, several cellular signaling pathways have been extensively described in the context of LLPS-mediated potentiation of animal viral innate immunity (Table 1), including the cGAS-STING-IRF3/IRF7 pathway, the dsRNA-PKR-eIF2α-SGs pathway, LLPS-triggered assembly of promyelocytic leukemia protein-nuclear bodies (PML-NBs), and LLPS-induced inflammasome formation.

LLPS augments the cGAS-STING pathway—one of the main pathways for cellular sensing of pathogenic DNA (Zhou et al., 2021; Yao et al., 2022), through increased cGAMP production and effective amplification of interferon signaling. In the same pathway downstream of STING, upon detection of the virus, IRF3/IRF7 will undergo LLPS to form nuclear condensates with DNA containing the interferon-stimulated response element (ISRE). This phase-separated state sequesters IRF7 in the nucleus in a manner to enhance the expression of type I interferon, thus promoting type I IFN (IFN-I) production (Qin et al., 2022). This cGAS-DNA condensate can protect DNA from degradation and enhance cGAS activation by excluding the exonuclease TREX1 (Yao et al., 2022), indicating that the antiviral innate immunity is enhanced by LLPS.

Studies on host-initiated LLPS are commonly observed in SGs, which are molecular condensates formed in response to various stresses (including viral dsRNA) and play a key role in regulating RLR signaling activation (Paget et al., 2023). Eukaryotic initiation factor 2α (eIF2α) phosphorylation is induced by the dimerization of the dsRNA-activated protein kinase R (PKR), which is expressed in most vertebrate cells and leads to SG formation (Pfaller et al., 2011; Matsumiya et al., 2023). Interestingly, recent studies have shown that SGs are not only a site for translation inhibition, but also serve as buffers that prevent excessive innate immune responses to dsRNA while maintain optimal interferon production. This suggests that SGs integrate immune signaling and cell survival decisions, making them an important component of the cellular response to viral infections (Matsumiya et al., 2023; Paget et al., 2023).

Apart from the cytosolic pathways, the nuclear PML-NBs also adopt LLPS to amplify antiviral responses (Matsumiya et al., 2023). PML-NBs are crucial for the antiviral defense, as they restrict viral replication and induce IFN production. The underlying mechanisms are mediated by recruitment of antiviral proteins (Everett and Chelbi-Alix, 2007; Wu et al., 2023). During infection with porcine reproductive and respiratory syndrome virus (PRRSV), the scaffold protein TRIM19 promotes the formation of PML-NBs in reliance of its α-helical CC domain, which drives the homomeric or heteromeric assembly of TRIM proteins, directly contributing to antiviral activity (Su et al., 2024). For pseudorabies virus (PRV) infection, swine PML (sPML)-NBs inhibit PRV replication by suppressing viral gene transcription. sPML contains two variants, best known as sPML-II and sPML-IIa. Their antiviral activity has been mapped to the 7b region, in which the cysteine residues 589 and 599 play critical roles in blocking the transcription of viral genes (Yu et al., 2020). Interestingly, all these processes are mediated by SUMOylation (Sahin et al., 2014; Corpet et al., 2020).

Beyond the cytosolic and nuclear pathways, LLPS also governs inflammasome assembly. Inflammasomes are caspase-activating cytoplasmic complexes of the innate immune response, which mediate pyroptosis and release of pro-inflammatory cytokines (e.g., IL-1β, IL-18) in response to infection or stress (Martinon et al., 2002). Their assembly is dependent on the cascade activation of caspase-1 by NLR sensors (i.e., NLRP3, NLRP6) that recognize danger signals (Liu et al., 2024). Particularly, NLRs control immunity through LLPS. NLRP6 undergoes LLPS with viral dsRNA to generate condensates that recruit apoptosis-associated speck-like protein containing a CARD (ASC) to facilitate caspase-1 activation and control of antimicrobial immunity. Viral infections (e.g., MHV, rotavirus) disrupt these condensates, leading to the inactivation of inflammasomes (Shen et al., 2021). p62-dependent phase-separated P-bodies (pd-PBs) mediate the formation of NLRP3 inflammasome and recruit ASC to enhance downstream signaling (Barrow et al., 2024). Collectively, LLPS exerts unique immunological effects on the regulation of inflammasomes, while NLRs are the key defenders for animal host to compete against viruses.

## 4 Viral targeting of immune condensates

**Figure 1:**
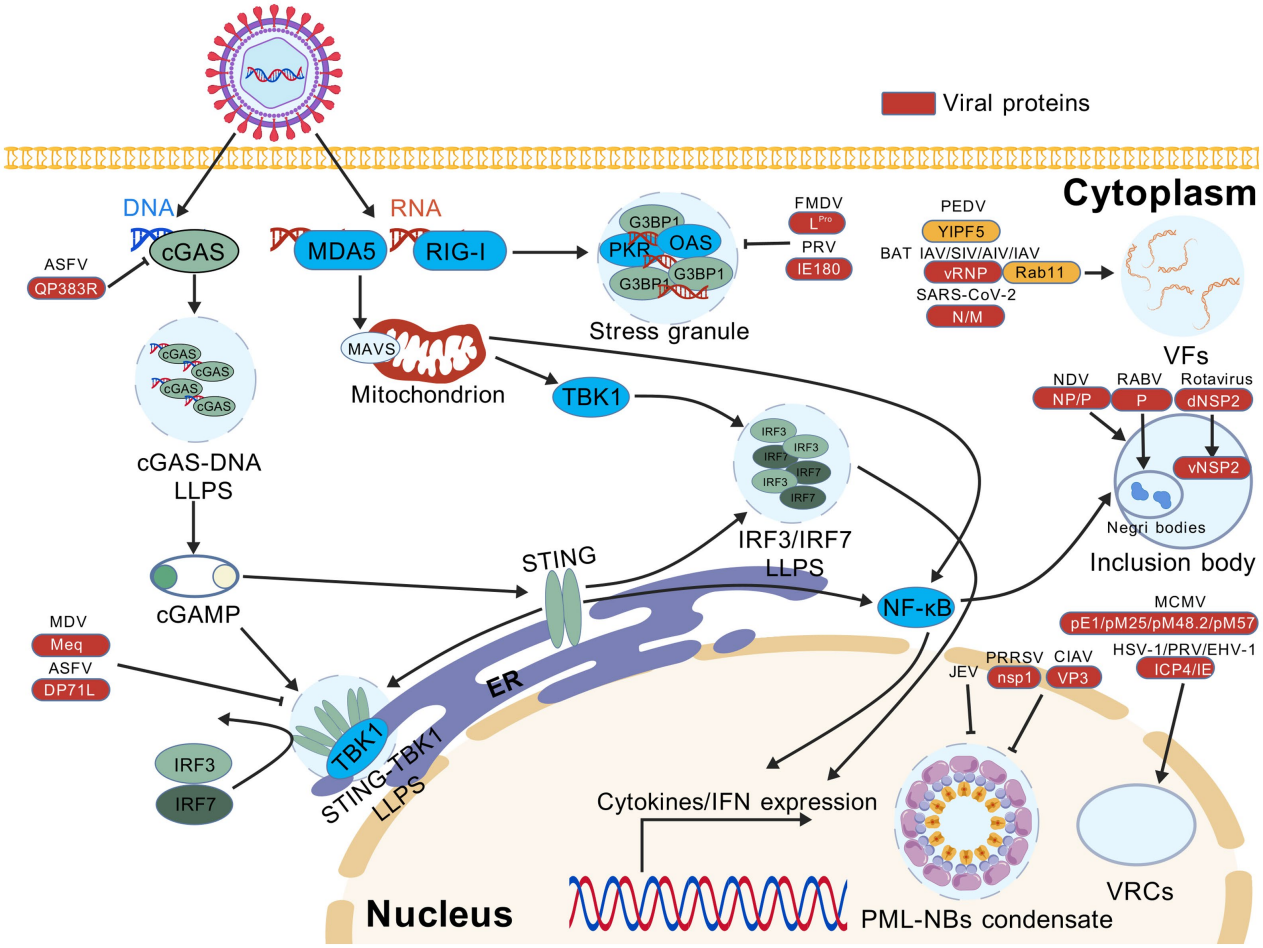
Viral proteins manipulate liquid–liquid phase separation (LLPS) to antagonize innate immune signaling pathways and promote virus replication. Cleavage of G3BP1 by foot-and-mouth FMDV and PRV suppresses the formation of stress granules (SGs) triggered by the RLR-mediated RNA sensing pathway. ASFV and MDV prevent the LLPS-mediated enrichment of DNA-binding cGAS and STING-TBK1 complex, leading to the impaired recognition of viral DNA by the cGAS-STING pathway. Infection with PRRSV, CIAV or JEV disrupts the formation of PML-NB condensates induced by LLPS. Furthermore, a variety of LLPS-associated mechanisms are required to support efficient virus replication. In the cytoplasm, viral factories (VFs) are formed by infection with many RNA viruses, e.g., SARS-CoV-2, AIV, SIV, BAT IAV, IAV, and PEDV, whereas NDV, rotavirus and RABV tend to induce the formation of inclusion bodies (IBs). In the nucleus, viral replication compartments (VRCs) are caused by the translocation of regulatory proteins in DNA viruses, e.g., PRV, EHV-1, HSV-1, and MCMV. AIV, avian influenza virus; ASFV, African swine fever virus; BAT IAV, bat influenza A virus; CIAV, chicken infectious anemia virus; EHV-1, equine herpesvirus type 1; FMDV, foot-and-mouth disease virus; HSV-1, herpes simplex virus type 1; IAV, influenza A virus; JEV, Japanese encephalitis virus; MCMV, mouse cytomegalovirus; MDV, Marek’s disease virus; NDV, Newcastle disease virus; PEDV, porcine epidemic diarrhea virus; PRV, pseudorabies virus; PRRSV, porcine reproductive and respiratory syndrome virus; RABV, rabies virus; SARS-CoV-2, severe acute respiratory syndrome coronavirus 2; SIV, swine influenza virus. This figure was created with BioGDP (under the agreement no. GDP20255B4TJF).

Animal viruses have evolved multifaceted strategies, including leveraging the virus-encoded proteins and host signaling pathways to manipulate or dismantle immune-related LLPS condensates for their benefits (Figure 1). The following sections will explore these mechanisms in details (Table 1).

### 4.1 Disruption of the cGAS-STING pathway

The DNA-specific sensor cGAS is a prime target for viral evasion. African swine fever virus (ASFV) utilizes virulence-associated proteins DP71L and QP383R to antagonize STING-dependent type I IFN signaling (Qi et al., 2023). DP71L contains a conserved PP1-binding motif that competes for the C-terminal tail (CTT) of STING and interferes with the STING-TBK1 interaction. Therefore, the presence of DP71L suppresses downstream phosphorylation events (e.g., STING, TBK1, and IRF3), ultimately destabilizing the LLPS of IRF3 required for antiviral IFN responses (Ranathunga et al., 2025). Concurrently, another viral factor QP383R impedes cGAS activity by inducing its palmitoylation, which blocks DNA binding, cGAS dimerization, and LLPS formation, thereby reducing cGAMP synthesis (Hao et al., 2023). Similarly, Marek’s disease virus (MDV) exploits its oncoprotein Meq to sterically prevent recruitment of TBK1 and IRF7 to the STING complex (Li et al., 2019), probably inhibiting LLPS of IRF7. In contrast, RABV adopts a resource competition strategy by which the viral N and P proteins redirect TLR3 for the strict biogenesis of Negri bodies as replication compartments (Ménager et al., 2009).

### 4.2 Inhibition of stress granules

To neutralize the dsRNA-PKR-eIF2α-SG axis, many viruses encode PKR inhibitors (Langland et al., 2006). Vaccinia virus (VACV) K3 protein exhibits host-specific suppression of PKR, strongly inhibiting bovine and murine PKR to enhance viral replication by reducing eIF2α phosphorylation, whereas these effects are absent in sheep, goats and humans (Park et al., 2019), suggesting that the co-evolutionary arms race between PKR and viral inhibitors is conserved among K3 orthologs that execute species-and virus-dependent antagonism of PKR (Park et al., 2021). In contrast to PKR targeting by DNA virus, RNA viruses directly modulate SG assembly via G3BP manipulation. Foot-and-mouth disease virus (FMDV) protease L^Pro^ cleaves G3BP1/2 to block SG formation (Visser et al., 2019), whereas NDV concurrently sort G3BP1 and TIA-1 into replication-promoting SGs that sequester host mRNA (Miao et al., 2023). Alternatively, PRV relocates G3BPs to the nucleus by its IE180 protein and causes the suppression of cytoplasmic SG assembly (Zhao et al., 2025), suggesting that hijacking nuclear transport might be commonly used by alphaherpesviruses for immune evasion. Similarly, the condensates formed by the SARS-CoV-2 N protein, M protein, and RNA can also recruit G3BP1 to inhibit SG formation, thereby suppressing the host innate immune response. The phosphorylation state of the N protein dictates its interaction with G3BP1 and the localization of host mRNA. Mechanistically, the condensates formed by unphosphorylated N protein or phosphorylation-resistant mutants N^14SA^ and N^ΔSR^ appear larger and denser, with stronger binding of host mRNA to these condensates (Lu et al., 2021). This suggests that post-translational modifications of the N protein are key in regulating viral replication and host immune suppression.

### 4.3 Destabilization of PML-NBs

Viruses of various animals have been shown to disrupt the LLPS-induced assembly of PML-NBs to enhance replication. The nonstructural protein nsp1 of PRRSV inhibits the activity of PML (Corpet et al., 2020), while apoptin also known as VP3 in chicken infectious anemia virus (CIAV) is modified by SUMO conjugation and targeted to PML-NBs (Janssen et al., 2007). In alphaherpesviruses (e.g., MDV, PRV), the US3 orthologs mediate PML-NB disruption through kinase-and proteasome-dependent mechanisms; but unlike the alphaherpesviral ICP0, they do not target PML for degradation (Jung et al., 2011; Liao et al., 2021). Japanese encephalitis virus (JEV) selectively destabilizes PML isoforms during infection, impairing antiviral immunity (Yang et al., 2023). In general, antagonism of LLPS-mediated intrinsic immunity is widespread in animal viral infections. In return, these evasion strategies highlight the critical role of LLPS in host antiviral defense, therefore further studies are warranted to understand how viruses exploit and disrupt phase-separated signaling hubs. Elucidating the molecular interplay between viral effectors and host phase-separated complexes will accelerate the development of LLPS-targeted antivirals and therapies.

## 5 Therapeutic engineering of the dynamics in phase separation

The ultimate goal of studying LLPS-based immune modulation ubiquitously observed in viral diseases is to develop therapeutic strategies. Through precise manipulation of LLPS, viral vectors can be modified to promote target gene expression, thereby achieving enhanced expression of antigens and development of gene therapies. As research into the LLPS phenomena and mechanisms is deepening, these concepts may become feasible. Some relevant speculations and ideas on the use of LLPS will be discussed as follows.

### 5.1 Therapeutic strategies via LLPS modulation

Highly active proteins appear to be the principal modulators of LLPS, thus pharmacological modification of target protein activity might provide an effective means of increasing resistance to disease, or alleviating pathology. For example, in the case of an alphaherpesvirus such as MDV, the treatment with the proteasome inhibitor MG132 partially restores PML bodies, which are disrupted by the viral US3 protein (Liao et al., 2021), indicating that the proteasome-dependent pathway can be therapeutically targeted and represent a viable strategy for counteracting the pathogenesis of alphaherpesviruses.

Mechanistically, cGAS boosts the efficiency of DNA uptake by the LLPS-guided formation of droplets (Yao et al., 2022). Immune modulators, such as IRTKS that reinforces the phase separation of STING, can potentiate interferon production (Xie et al., 2024). Additionally, the PI3K and MAPK/p38 pathways are engaged in promoting SG assembly through the mechanistic target of rapamycin complex 1 (mTORC1), making them potential therapeutic antiviral targets (Heberle et al., 2019). A notable feature of LLPS driven by the RABV phosphoprotein is dependent on its thermo-responsive property, wherein temperature and ionic strength regulate the LLPS state (Bouchama et al., 2025), presenting a therapeutic avenue for the treatment of localized hyperthermia. For the rapidly evolving RNA viruses, the effectiveness of LLPS-based therapeutic approaches largely depends on the structural conservation of targeted proteins and the binding affinity of sequence-specific genomic loci. It has been recognized that evolutionary mutations are unevenly distributed in the genomes of RNA viruses (Kustin and Stern, 2021). If the LLPS-based antivirals are designed to target the viral glycoproteins for cellular entry and egress, in which higher frequency of sequence mutations tend to occur, their inhibitory effects will be considerably diminished. In contrast, sequences coding for RNA-dependent RNA polymerase (RdRp) and other nonstructural proteins are relatively conserved in the species of a closely related family, and thus targeting these most conserved regions is expected to favor the benefit of LLPS-driven antiviral therapeutics.

### 5.2 Optimization of viral vectors based on LLPS principles

The improvement of LLPS properties through capsid engineering provides great potential for viral vector optimization. PRV, for instance, hijacks host protein-mediated LLPS for nuclear entry (Zhao et al., 2025). A central question is whether the engineered capsids can target low-complexity domains (LCDs) or nuclear VRCs to increase the efficiency of transduction. The use of such vectors would greatly benefit from engineering viral capsids to take advantage of IDR-mediated LLPS to fine-tune the efficiency of the vector. Current siRNA studies have evaluated interactions between nucleolar proteins (B23/nucleophosmin and nucleolin) and adeno-associated virus type 2 (AAV2) capsids. Similar to inhibition of proteasome, knockdown of nucleophosmin enhanced the nuclear accumulation of AAV2 and increased its transduction efficiency by 5–15 folds (Johnson and Samulski, 2009). Given the conserved mechanisms of nuclear transport between AAV and PRV, these findings may provide transferable strategies to engineer PRV vectors. In addition, mRNA can modify T cells through chimeric antigen receptor (CAR)-T cells, and LLPS-mediated antigen aggregation can enhance T cell responses (Wiesinger et al., 2019; Lee et al., 2023). This may suggest that future mRNA-lipid nanoparticle (LNP) vaccines could target LLPS structures to exert clinical potential.

## 6 Concluding remarks and future directions

Phase separation has emerged as one of the mechanistic insights into how virus and host immune system strive for the limited intracellular resource to fight against each other. The roles of LLPS are exerted by driving the formation of biomolecular condensates, which not only help to combat infections but may also promote viral replication and proliferation. Although LLPS is less studied in veterinary or zoonotic viruses compared to the human counterparts, the underlying mechanisms of phase separation provide a distinct perspective on the interplay between viruses and host cells, offering promising antiviral strategies.

Currently, no effective approach is available to directly control the outcome of LLPS-induced cellular processes, because the formation of biomolecular condensates is dynamically altered and intertwined with many signaling nodes. However, the discovery that metal ions specifically regulate the functions of IDRs might open up a new horizon. The IDR-containing protein SmbP from the ammonia-oxidizing bacterium *Nitrosomonas europaea* binds to bivalent cations (especially copper) to prevent cytotoxicity (Barney et al., 2004). Since IDRs are devoid of stable and targetable three-dimensional confirmations, a potential solution will rely on the use of CRISPR or RNAi technology to enhance the functions of enzymes associated with IDR stability (Kim et al., 2024). Additionally, targeted degradation strategy can also be useful. For example, chemical compounds targeting IDRs have been shown to inhibit the replication of human papillomavirus (HPV) genome by disrupting the binding of phosphorylated BRD4 with DNA damage response factors (Wu et al., 2024). Rather than stabilization of IDR-containing proteins, broader therapeutic targets can be made by self-regulation of LLPS. For instance, the inhibitor elvitegravir disrupts the formation of SRC-1 condensates in cancer cells. This inhibition holds promise for the LLPS-based strategy as therapeutic option for treating the YAP-dependent cancer (Zhu et al., 2021).

Structurally designed small molecules will also be powerful candidates to destroy the LLPS-induced compartments. Cyclopamine (CPM) inhibits RSV replication by toughening and shrinking IBs *in vivo*. Moreover, both CPM and its chemical analog aethoxydiol (A3E) can modulate the activity of transcription factors by targeting the M2-1 protein, thereby hardening condensates and enhancing antiviral effects (Risso-Ballester et al., 2021). Other noticeable candidates are PARP inhibitors. Among them, PJ34 and CVL218 act as buffering molecules that reduce the local density of condensates, increase permeability, and enhance drug utilization, which ultimately lead to dissolution of condensates (Zhao et al., 2021). In contrast, another PARP inhibitor olaparib can regulate condensate formation and the transport of their components (Düster et al., 2021). Although 1,6-hexanediol is widely used for LLPS dissolution, its detrimental effects on kinase and phosphatase activity make it difficult to be the optimal drug for targeting phase-separated condensates (Düster et al., 2021). Therefore, the structures of small-molecule inhibitors need to be carefully designed to avoid prospective off-targe effects by using artificial intelligence (AI)-based screens.

At present, precise manipulation of LLPS to inhibit viral replication but to avoid immune suppression remains extremely challenging. This requires comprehensive deciphering of the regulatory mechanisms that engage different biological functions of a given molecule. Selection of targets should focus on the critical amino acid residues or nucleotide sequences that determine both the formation of LLPS-induced viral replication compartments and the signaling transduction events of immune responses. The desired effects can first be evaluated by AI-powered weighted analysis models, and then achieved by synthesis of biologically active molecules (e.g., peptides or oligomers) that interfere with LLPS processes. Rather than a single target, integration of multiple potential ones might help mitigate the risk of unanticipated effects caused by modulating LLPS.

Research on LLPS in veterinary viruses is still in its infancy, and particularly the immunological significance of protein condensates induced by LLPS remains largely unclear. Inside a cell, a majority of proteins are able to undergo LLPS, but their diversity and complexity are an intrinsic problem when a broad array of cellular events are associated with biomolecular condensates. To address this conundrum, ultra-high-resolution electron microscopy and sophisticated molecule-tracking techniques are required. Because the study of LLPS has become multidisciplinary, veterinary virologists interested in this area must be trained to integrate knowledge and expertise of materials science, biophysics and cell biology. With increased understanding of how viruses hijack LLPS for their persistence and immune evasion, more effective and targeted antivirals or therapeutics will be developed to prevent the enormous losses of domestic animals caused by viral infections.
